# Assessing blood-brain barrier dysfunction and its association with Alzheimer’s pathology, cognitive impairment and neuroinflammation

**DOI:** 10.1186/s13195-024-01529-1

**Published:** 2024-07-31

**Authors:** Lukas Preis, Kersten Villringer, Frederic Brosseron, Emrah Düzel, Frank Jessen, Gabor C. Petzold, Alfredo Ramirez, Annika Spottke, Jochen B. Fiebach, Oliver Peters

**Affiliations:** 1 Charité – Universitätsmedizin Berlin, corporate member of Freie Universität Berlin and Humboldt-Universität zu Berlin-Institute of Psychiatry and Psychotherapy, Berlin, Germany; 2 Centre for Stroke Research Berlin, Charité – Universitätsmedizin Berlin, corporate member of Freie Universität Berlin and Humboldt-Universität zu Berlin, 12200 Berlin, Germany; 3 German Center for Neurodegenerative Diseases (DZNE), Bonn, Venusberg-Campus 1, 53127 Bonn, Germany; 4 Department for Cognitive Disorders and Old Age Psychiatry, University Hospital Bonn, Bonn, Germany; 5 German Center for Neurodegenerative Diseases (DZNE), Magdeburg, Germany; 6 Institute of Cognitive Neurology and Dementia Research (IKND), Otto-von-Guericke University, Magdeburg, Germany; 7 Department of Psychiatry, Medical Faculty, University of Cologne, Kerpener Strasse 62, 50924 Cologne, Germany; 8 Excellence Cluster on Cellular Stress Responses in Aging-Associated Diseases (CECAD), University of Cologne, Joseph-Stelzmann-Strasse 26, 50931 Köln, Germany; 9 Center for Neurology, University of Bonn, Venusberg-Campus 1, 53127 Bonn, Germany; 10 Division of Neurogenetics and Molecular Psychiatry, Department of Psychiatry and Psychotherapy, Faculty of Medicine and University Hospital Cologne, University of Cologne, Cologne, Germany; 11 Department of Psychiatry & Glenn Biggs Institute for Alzheimer’s and Neurodegenerative Diseases, San Antonio, TX USA; 12 German Center for Neurodegenerative Diseases (DZNE), Berlin, Germany

**Keywords:** Alzheimer’s disease, Mild cognitive impairment, Blood-brain barrier, Pericyte, sPDGFRβ, DCE-MRI

## Abstract

**Background:**

Blood-brain barrier (BBB) alterations may contribute to AD pathology through various mechanisms, including impaired amyloid-β (Aβ) clearance and neuroinflammation. Soluble platelet-derived growth factor receptor beta (sPDGFRβ) has emerged as a potential biomarker for BBB integrity. Dynamic contrast-enhanced magnetic resonance imaging (DCE-MRI) offers a direct assessment of BBB permeability. However, the relationship between BBB dysfunction, cognitive impairment, and AD pathology remains unclear, with inconsistent findings in the literature.

**Methods:**

We conducted a cross-sectional study using data from the DELCODE and DESCRIBE cohorts to investigate BBB dysfunction in participants with normal cognition (NC), mild cognitive impairment (MCI), and AD dementia. BBB function was assessed using DCE-MRI and sPDGFRβ levels in cerebrospinal fluid and AD biomarkers Aβ and tau were measured. In a subset of patients, the CSF/plasma-ratio of albumin (QAlb) as a standard marker of BBB integrity and markers of neuroinflammation were analyzed.

**Results:**

91 participants (NC: 44, MCI: 21, AD: 26) were included in the analysis. The average age was 74.4 years, 42% were female. Increased hippocampal BBB disruption was observed in the AD-group (K^trans^: 0.55 × 10^− 3^ min^− 1^ ± 0.74 × 10^− 3^ min^− 1^) but not the MCI-group (K^trans^: 0.177 × 10^− 3^ min^− 1^ ± 0.22 × 10^− 3^ min^− 1^), compared to the NC group (K^trans^: 0.19 × 10^− 3^ min^− 1^ ± 0.37 × 10^− 3^ min^− 1^, *p* < .01). sPDGFRβ was not significantly different between the cognitive groups. However, sPDGFRβ levels were significantly associated with age (*r* = .33, *p* < .01), independent of vascular risk factors. Further, sPDGFRβ showed significant positive associations with soluble Aβ levels (Aβ40: *r* = .57, *p* < .01; Aβ42: *r* = .39, *p* < .01) and YKL-40 (*r* = .53, *p* < .01), a marker of neuroinflammation. sPDGFRβ/DCE-MRI was not associated with overall AD biomarker positivity or APOE-status.

**Conclusion:**

In dementia, but not MCI, hippocampal BBB disruption was observed. sPDGFRβ increased with age and was associated with neuroinflammation independent of cognitive impairment. The association between Aβ and sPDGFRβ may indicate a bidirectional relationship reflecting pericytes’ clearance of soluble Aβ and/or vasculotoxic properties of Aβ.

**Supplementary Information:**

The online version contains supplementary material available at 10.1186/s13195-024-01529-1.

## Background

In 2018 the NIA-AA introduced a biological classification for Alzheimer’s disease (AD) based upon the AT(N)-framework that includes amyloid-pathology, tau-pathology and neurodegeneration [1]. Recognizing advancements in our understanding of AD, an extension of the AT(N)-classification (ATXN) was proposed to include additional pathophysiological mechanisms of the disease [2]. Blood-brain barrier (BBB) changes may be one relevant pathophysiological domain to consider. The BBB represents the interface between the central nervous system (CNS) and the peripheral blood-circulation and is part of the neurovascular unit consisting mainly of endothelial cells, pericytes and astrocytes [3]. It protects the brain from external pathogens as well as inert neurotoxic substances (e.g. fibrinogen) to enter the CNS [4]. It also maintains homeostasis in the brain by expressing a multitude of receptors, ion-channels, and specific-transport systems, thereby providing for the brain’s large energy demand, but also serving as one of the brain’s critical clearance systems [4].

As proposed by the ‘two-hit vascular hypothesis’ [5] of AD, BBB dysfunction may be a potential early contributor to amyloid-β (Aβ) accumulation, because, to a large extent, Aβ is cleared out of the brain across the BBB [4, 6, 7]. Reduced clearance capabilities may therefore predispose for the built-up of Aβ plaques. Beyond amyloid-dependent disease mechanisms, BBB dysfunction may also induce neurodegenerative processes via the infiltration of neurotoxic substances across a deficient BBB [8], lead to neuroinflammation [9], and cause pericyte-mediated cerebral hypoperfusion [10].

To measure the function of the BBB, the ratio of albumin in the cerebrospinal fluid (CSF) and plasma (QAlb) is routinely used in clinical practice. The liver is the sole organ capable of synthetizing albumin and no active transport mechanisms across the BBB have been described making it a suitable candidate to assess BBB integrity [11]. However, the molecular weight of albumin of 66.5 kDA [12] is rather large questioning its appropriateness to detect minor paracellular BBB leakage. Accordingly, Kurz and colleagues [13] report it’s diagnostic sensitivity for subtle BBB changes in the context of AD to be limited with some studies demonstrating increased QAlb in patients with dementia [14, 15] but not consistently in mild cognitive impairment (MCI) [15, 16].

Soluble platelet-derived growth factor receptor beta (sPDGFRβ), a receptor that is expressed on pericytes [17] has been proposed as a novel and sensitive biomarker of BBB disruption [18]. Pericytes can be found adjacent to capillary endothelial cells and are thereby part of the neurovascular unit [4]. Through constriction they can regulate capillary blood-flow [19], clear Aβ out of the brain [6, 20] and are crucial for maintaining overall BBB integrity [21, 22]. Though sPDGFRβ is not exclusively expressed by pericytes but also by vascular smooth muscle cells, Sagare and colleagues [17] have shown that only pericytes shed sPDGFRβ into the CSF in response to noxious stimuli. sPDGFRβ may thereby serve as a biomarker of pericyte degeneration and a proxy for BBB integrity. Another method, known as dynamic contrast-enhanced magnetic resonance imaging (DCE-MRI) [23], offers an alternative approach to assess BBB function. DCE-MRI provides a means to directly visualize and quantify the permeability of the BBB in specific brain regions. This technique utilizes the transfer constant (K^trans^) of a paramagnetic contrast agent which induces T1-shortening, allowing for the measurement and quantification of extravasation into the brain parenchyma [24].

Clinical studies investigating BBB function using DCE-MRI showed age-dependent increases in K^trans^ in the hippocampus that rose in patients with cognitive decline [25]. Hippocampal BBB disruption occurred independent of typical AD biomarkers [18] but was associated with APOE4 [26]. Regarding the literature on sPDGFRβ, some studies have shown that sPDGFRβ was increased in AD [27] and linked to APOE4-status [26], other studies reported an association with cognitive dysfunction irrespective of AD-pathology [18]. A recent study by Cicognola and colleagues [28] revealed opposing results in a large and well characterized cohort. This study identified age-dependent effects on sPDGFRβ and associations with neuroinflammation but no association with AD-biomarkers, APOE4 or cognitive decline.

Considering these discrepancies throughout the literature, the aim of this study is to investigate BBB dysfunction in patients suffering from MCI and AD-dementia, compared to participants with normal cognition (NC), using data from the DELCODE [29] and DESCRIBE cohort (German Center for Neurodegenerative Diseases; DZNE). We quantified BBB function in the hippocampus using DCE-MRI and by measuring sPDGFRβ in the CSF in a subset of patients. We attempt to validate the association of sPDGFRβ/DCE-MRI to BBB dysfunction using QAlb and study the association of BBB dysfunction and routinely used AD-biomarkers in the CSF (i.e. amyloid-beta, tau). Lastly, we explore possible associations between BBB changes and a large panel of neuroinflammatory CSF biomarkers that were measured in DELCODE and previously linked to neurodegenerative processes in AD [30].

## Methods

### Ethical approval and patient consents

The research received ethical approval from the Ethics Commission of Charité University Medicine Berlin (Local ethics approval number: EA4/136/19, ClinicalTrials-gov ID: NCT04093882). Each participant provided written consent after being informed about the study procedures. The study was conducted in accordance with the Declaration of Helsinki.

### Study cohort

The individuals involved in this research were recruited from two observational cohorts ‘DELCODE’ (*n* = 71) and ‘DESCRIBE’ (*n* = 30), both allocated to the DZNE. As DCE-MRI measurements are not part of DELCODE or DESCRIBE we adopted a monocentric approach for this research project. For a detailed description of the study cohort see Jessen and colleagues [29].

Participants were clinically assessed using the clinical dementia rating [31] (CDR) and neuropsychometrically examined using the CERAD + test battery [32], which included the Mini-mental State Examination (MMSE) [33]. Depressive symptoms were measured using the geriatric depression scale (GDS) [34]. Vascular risk factors were determined through interviews and medical reports. Vascular risk factors considered were arterial hypertension, dyslipidemia, diabetes, history of vascular event (i.e., stroke, TIA, peripheral artery disease) and cardiac arrhythmias. Participants with two or more cardiovascular risk factors were considered as having a risk profile analog to Nation and colleagues [18].

Participants were approached to take part in the study during their annual visit that included the clinical examination and neuropsychometric testing. All participants who did not meet pre-defined exclusion criteria were approached to take part in the study. We included participants diagnosed with MCI, AD-dementia and individuals that showed no cognitive impairment (NC). The diagnosis of AD-dementia was established in accordance with the NIA-AA criteria [1]. MCI was diagnosed based on a clinical dementia rating (CDR) global score of 0.5 and evidence of cognitive dysfunction in the CERAD + test battery. The NC group demonstrated full cognitive proficiency in the CERAD + test battery. A cognitive deficit in the CERAD + was defined by a test score falling 1.5 standard deviations below norms adjusted for age and education.

Neurodegenerative diseases other than AD and psychiatric disorders significantly affecting cognition were excluded. Furthermore, we excluded subjects with extensive cerebral small-vessel disease and subjects with conditions resulting in (sub)acute blood-brain barrier dysfunction (e.g. acute stroke).

### CSF sampling and biomarker analysis – sPDGFRβ

CSF was collected with polypropylene tubes and stored at -80 °C. Aβ42, Aβ40, p(181)tau, and total tau were measured using the fully-automated Fujirebio-LUMIPULSE G600II (Fujirebio Holdings Inc., Tokyo, Japan) system using the dedicated immunoreaction cartridges. To measure sPDGFRß we used the „Human PDGFR beta ELISA Kit“ by ThermoScientific© (Thermo Fisher Scientific, Waltham, United States) according to the specified manual. Albumin in plasma and CSF was measured using the BN™ II System by Siemens (Erlangen, Germany), a fully automated nephelometric analyzer. The panel of candidate neuroinflammatory CSF biomarkers consisted of micro- and astroglial markers (sTREM2, YKL-40), cytokines and chemokines (MCP-1, IP10, MIF, IL-6, IL-18, CRP), immune-regulating receptors of the TAM signaling pathway (sAXL, sTyro3) and complement factors C1q, C3, C3b, C4, Factor B and H. This panel was established in previous works by Brosseron and colleagues [30–36]. In brief, this panel contains well quantifiable markers of different inflammation-associated pathways (such as phagocytosis, complement, pro-inflammatory mediators and regulation of inflammation). The panel furthermore focusses on markers with association to tau isoform levels and markers of neurodegeneration, as described in referenced studies. In CSF, markers of the TAM pathway were also related to preserved structure and cognition. The markers were originally measured from aliquoted CSF samples by a series of immunoassays utilizing different detection techniques (e.g., colorimetric, electrochemiluminescence, bead-based, and single molecule tray / SIMOA). Each assay was optimized to the different abundance and quantitation range of high abundant proteins like YKL-40, or low abundant proteins like IL-6 in CSF. Samples were determined in duplicates with a maximum accepted variance of 20%. Complete method details on assay and dilution have been described in the referenced studies.

### DCE-MRI

Images were acquired on a 3T Prisma fit MRI scanner (Siemens Healthineers, Erlangen, Germany) using a 64-channel head coil. Cerebral small vessel disease was assessed by neuroradiologists blinded to the patients’ diagnosis using the age-related white matter change score (ARWMC) [37].

The T1 dynamic protocol comprised pre-contrast T1 measurements with four different flip angles (2°, 10°, 20°, 35°) for T1 mapping. TE (echo time) = 2.9 msec, TR (repetition time) = 60 msec, FOV 220, voxel size 1.7 × 1.7 × 2.5 mm, 9 slices orientated alongside the hippocampus, slice thickness 2.5 mm without gap. T1 mapping was followed by the DCE protocol in form of a continuous serial acquisitions of 60 volumes of T1-weighted images. Ten mL Gadovist (Gd) (Gadobutrol, 1 M, Bayer Schering Pharma AG, Berlin, Germany) at a flow rate of 1 mL/s was continuously administered intravenously 1 min after start of the acquisition, followed by a 20 mL saline flush. The imaging parameters for T1 measurements were as follows: TE = 2.5 ms, TR = 50 ms, 9 slices, 2.5 mm slice thickness without gap, flip angle 60°. FOV and voxel size were the same as in the T1 mapping protocol. The arterial input function was selected in the internal carotid artery in the cavernous segment. The total scan time amounted to about 7 min.

Postprocessing of data included motion correction (FLIRT = FMRIB’s linear image registration tool, FSL, FMRIB, Oxford, UK, (https://fsl.fmrib.ox.ac.uk/fsl/)), followed by regions of interests (ROIs) manually drawn on the hippocampus on all slices where visible on T1 structural images. For BBB assessment the open source software package ROCKETSHIP (https://github.com/petmri/ROCKETSHIP) [38] was employed using the Patlak model. The quantification of the BBB was performed for the ROI covering the whole hippocampus once for the right and once for the left side. The derived metric for capillary permeability is referred to as the transfer constant K^trans^.

### ApoE4

The genotyping of the rs7412 and rs429358 genotypes, which determine the ε-2, ε-3, and ε-4 alleles of the APOE gene, was conducted using the TaqMan^®^ SNP Genotyping Assay from ThermoFisher Scientific (Waltham, United States), a commercially available kit. Both SNP assays were amplified from genomic DNA utilizing the StepOnePlus Real-Time PCR System, also from ThermoFisher Scientific. Prior to utilizing the genotype data to characterize the ε-2, ε-3, and ε-4 alleles in each sample, a visual examination of cluster formation was performed for each SNP.

### Statistical analyses & study design

This is a cross-sectional, prospective study design. Data analysis was performed using SPSS version 29 [39], visualization of the data was performed using the ‘ggplot2’-package [40] within R-Studio version 4.3.1 [41]. Statistical assumptions for parametric testing procedures, such as homoscedasticity and normality of the residuals were ensured before analysis. Log10-transformations were applied to all biomarkers including K^trans^ values to fulfill statistical assumptions. For the DCE-MRI analysis we excluded 10 participants due to extensive white matter disease (ARWMC > 9) that was not identified before study inclusion. We explored quantitative measures to assess implausibility without implementing any specific cut-off criteria for excluding outliers.

**Fig. 1:**
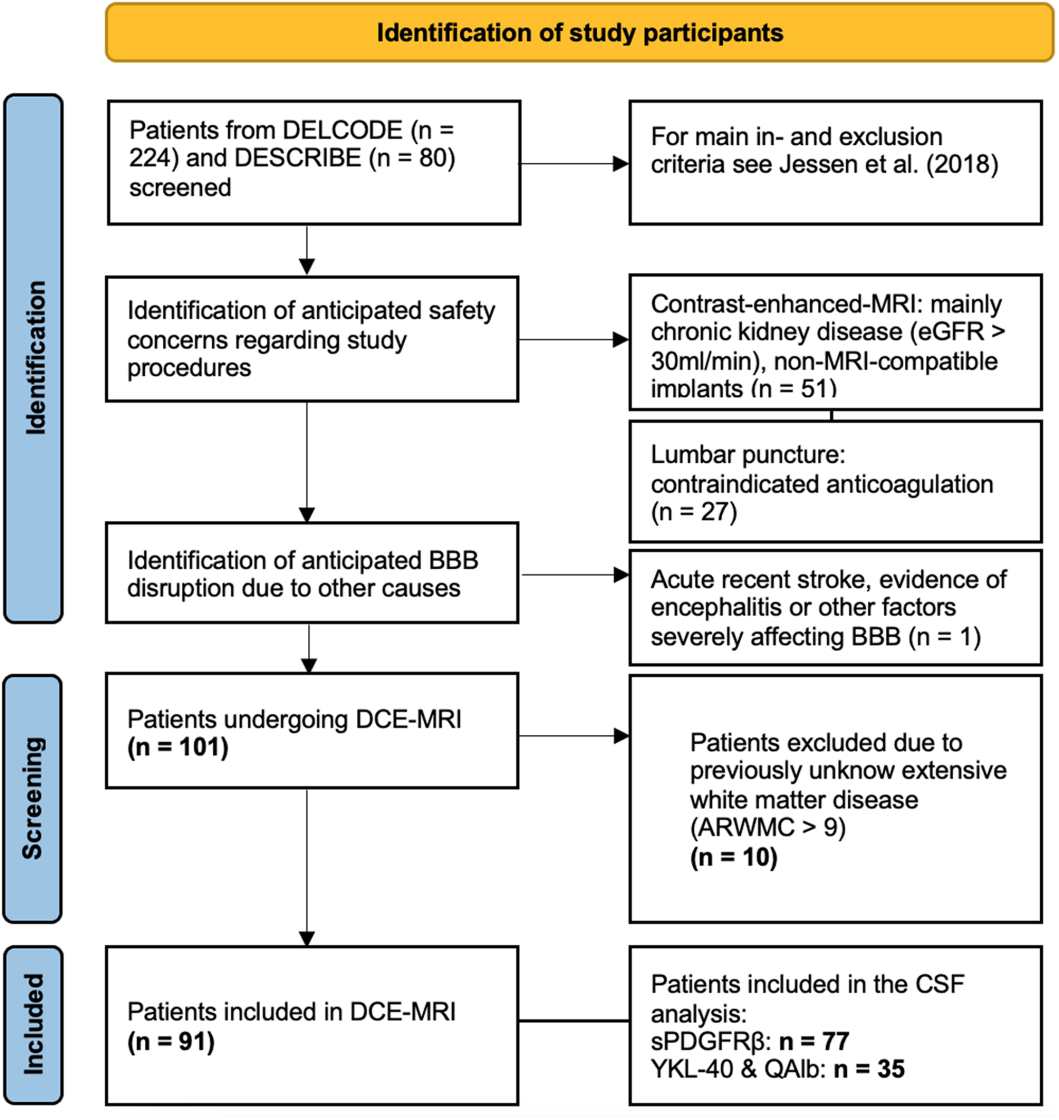
Flow chart: participant exclusion Abbrevations: DCE-MRI = dynamic contrast-enhanced MRI; ARWMC = age-related white matter changes; CSF = cerebrospinal fluid; sPDGFRβ = soluble platelet-derived growth factor receptor β; QAlb = Albumin ratio; YKL-40 = chitinase 3-like protein 1; eGFR = estimated glomerular filtration rate

CSF samples from 77 individuals were accessible for sPDGFRβ analysis. Markers of neuroinflammation were only assessed within the DELCODE-study as part of an overarching multicentric investigation (*n* = 35). Accordingly, markers of neuroinflammation were assessed during participants’ baseline study-visit, resulting in larger time intervals in between the biomarker assessments. On average, markers of neuroinflammation were assessed 2 to 5 years before markers of BBB-integrity. For an overview of patient selection and exclusion see Fig. 1.

Group comparisons were performed using univariate analysis of covariance (ANCOVA). Main models were controlled for age, sex, APOE4-status, and vascular risk factors where applicable. Linear associations were investigated with Pearson’s correlation and hierarchical multiple linear regression with covariates applied in the first block where applicable. To explore significant associations between markers of BBB integrity and markers of neuroinflammation a correlation matrix was used (Supplementary materials Figure S1). Bonferroni’s method was applied to address for multiple significance-testing.

## Results

### Study cohort

In the full cohort, most participants (*n* = 44) had normal cognition, 21 participants were classified as MCI, and 26 diagnosed with AD-dementia. The average age was 74.4 years, 42% were females and the average years of education were 14.7. One third of the participants had one vascular risk factor, 29% had two or more vascular risk factors. The most common vascular risk factor was arterial hypertension. Cerebrovascular white matter disease was mild (ARWMC = 6 ± 4.1). Participants diagnosed with MCI had MMSE scores averaging 28.2 (± 1.6), in contrast to the AD dementia group, which exhibited lower MMSE scores averaging 22.3 (± 4.0). CDR sum of boxes revealed mild deficits in activities of daily living in the MCI group (1.9 ± 1.1) and more pronounced deficits in the AD dementia group (5.0 ± 1.7). Using locally validated cut-off scores (Aβ42 < 680 pg/ml, Aβ42/40 < 0.055, Tau 400 X pg/ml, p(181)Tau > 62 pg/ml), almost all participants with AD-dementia showed abnormalities in Aβ40/42-ratio, compared to approximately 50% of all participants in the MCI-group and around 20% in the NC-group. Regarding tau pathology we observed abnormalities in 40% in the NC group, 55% in the MCI group and 96% in the AD group. Similar results were observed for p(181)tau (Table 1).

**Table 1:** Demographics and clinical characteristics

	NC (*n* = 44)	MCI (*n* = 21)	AD (*n* = 26)	*P* value
Age (years)	73.3 (5.8)	74.5 (7.0)	74.5 (8.3)	0.68
Sex (f; %)	14 (32%)	9 (43%)	15 (58%)	0.11
Education	15.5 (2.9)	15.1 (3.1)	13.8 (3.4)	0.10
Vascular risk factors	1.1 (1.2)	1.0 (1.2)	0.7 (0.7)	0.25
MMSE	**29.2 (0.8)**	**28.2 (1.6)**	**22.3 (4.0)**	**< 0.001**
CDR-SB	**0.3 (0.4)**	**1.9 (1.1)**	**5.0 (1.7)**	**< 0.001**
ARWMC	4.7 (1.9)	4.6 (2.1)	5.7 (2.0)	0.09
Aβ40/42	**0.090 (0.029)**	**0.068 (0.029)**	**0.048 (0.014)**	**< 0.001**
p(181)tau (pg/ml)	**54.5 (22.6)**	**65.3 (20.1)**	**106.0 (37.1)**	**< 0.001**
total tau (pg/ml)	**359.3 (133.1)**	**401.1 (153.6)**	**675.4 (206.0)**	**< 0.001**
DCE-MRI K^trans^ (10^− 3^ min^− 1^)	**0.19 (0.37)**	**0.17 (0.22)**	**0.55 (0.74)**	**0.007**
sPDGFRβ (pg/ml)	176.55 (53.15); *n* = 44	149.25 (29.01); *n* = 18	162.80 (46.80); *n* = 15	0.20
QAlb	5.81 (1.99); *n* = 13	5.95 (1.37); *n* = 10	5.39 (2.30); *n* = 10	0.84

Note. Depicted are means and standard deviations in brackets, sex is depicted in percentages

Abbreviations: MMSE = mini mental state examination; CDR-SB = clinical dementia rating sum of boxes; ARWMC = age-related white matter changes; DCE-MRI = dynamic contrast-enhanced magnetic resonance imaging; sPDGFRβ = soluble platelet-derived growth factor receptor-β; QAlb = Albumin ratio; Aβ = Amyloid-beta

### Aging, vascular risk factors, QAlb and sPDGFRβ/DCE-MRI

**Fig. 2:**
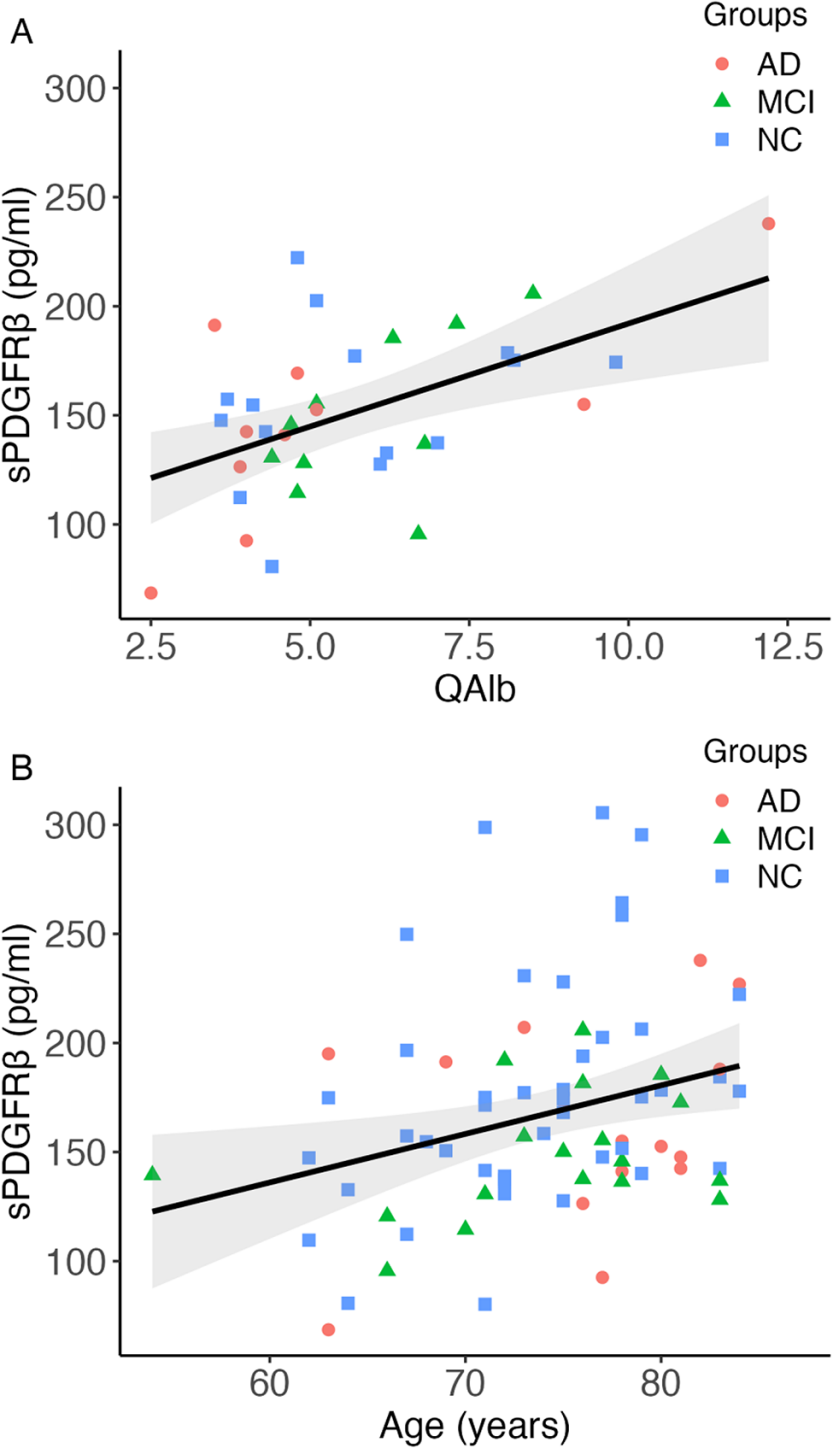
Association between sPDGFRβ with QAlb and age **A** Scatterplot depicting a significant positive correlation between sPDGFRβ and QAlb (*r* = 0.49, *p* < .01) **B** Scatterplot depicting a significant positive correlation between sPDGFRβ and age (*r* = 0.33, *p* < .01) Abbrevations: sPDGFRβ = soluble platelet-derived growth factor receptor β; QAlb = Ratio of albumin in cerebrospinal fluid and blood

Higher sPDGFRβ (*r* = .51, *p* < .01; Fig. 2a) but not DCE-MRI K^trans^ values (*r*=-.10, *p* = .57) were associated with increased QAlb. This association remained significant when controlling for age and sex (*b* = 0.27, β = 0.33, *p* = .049). sPDGFRβ and DCE-MRI did not significantly correlate (*r*=-.21, *p* = .07; Supplementary materials Figure S2A).

Aging was associated with increased sPDGFRβ (*r* = .34, *p* < .01) and remained a significant predictor after controlling for sex, APOE4-status, and vascular risk factors (*b* = 0.007, β = 0.368, *p* = .001; Fig. 2b). sPDGFRβ was not associated with white matter hyperintensities (*r* = .001, *p* = .99; Supplementary materials Figure S2B) or an increased vascular risk profile (*p* = .98). K^trans^ did not show associations to aging (*r* = .004, *p* = .97; Supplementary materials Figure S2C), white matter hyperintensities (*r*=-.7, *p* = .49; Supplementary materials Figure S2D) or vascular risk profile (*p* = .91).

### Cognitive dysfunction and sPDGFRβ/DCE-MRI

**Fig. 3:**
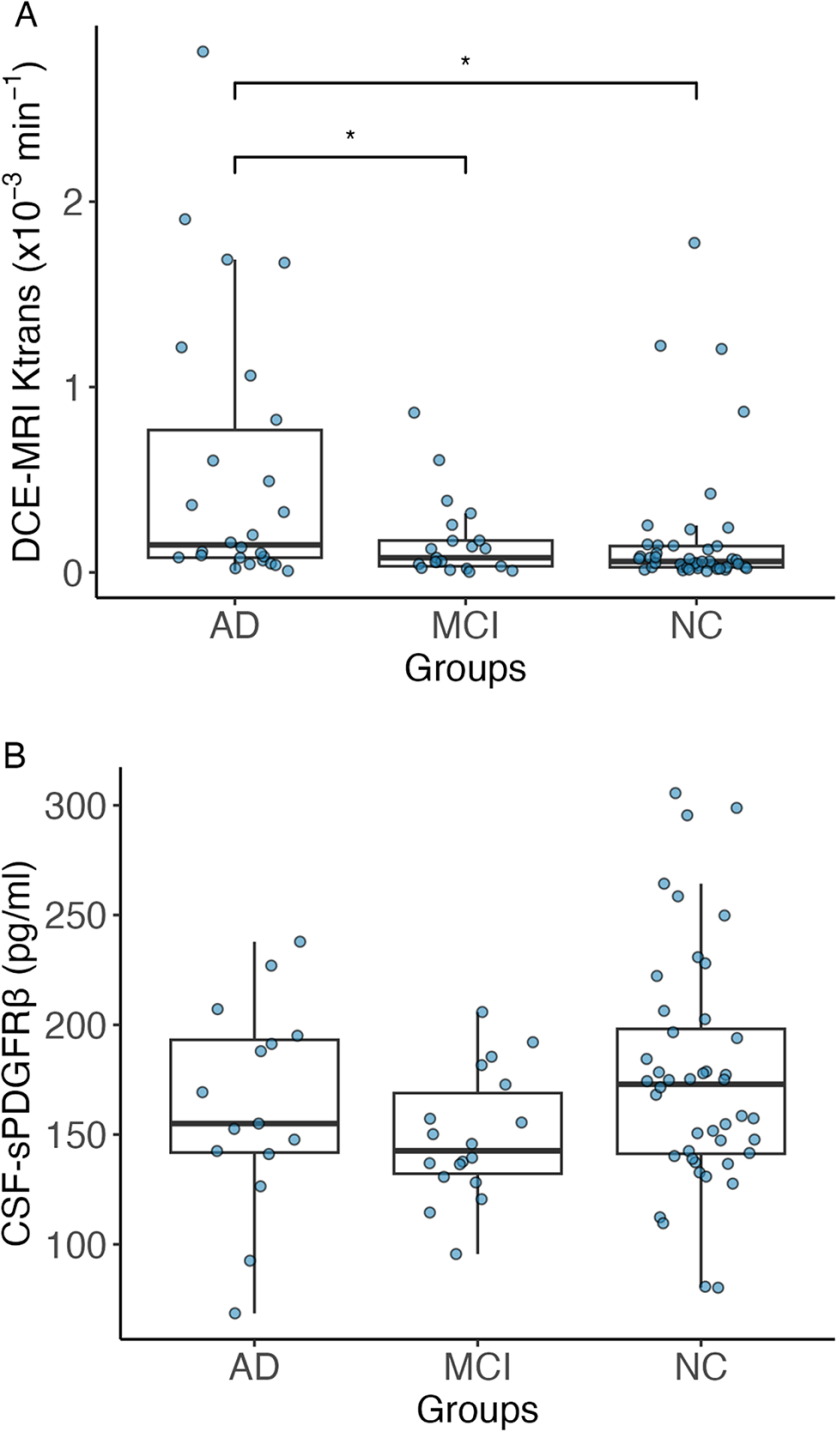
ANCOVA analysis of DCE-MRI and sPDGFRβ between NC, MCI and AD. **A** Boxplot indicating significant differences between the NC group and AD group (*p* < .01) and between the MCI and AD group (*p* = .03). **B** Boxplot indicating no significant difference in sPDGFRβ between the groups (ANCOVA) Abbreviations: sPDGFRβ = soluble platelet-derived growth factor receptor β; DCE-MRI = dynamic contrast-enhanced magnetic resonance imaging; NC = normal cognition; MCI = mild cognitive impairment; AD = Alzheimer’s dementia

Analysis of variance revealed significant differences (*p* < .01) in K^trans^ between the cognitive groups. This difference remained significant when controlling for age, sex, APOE4-status and vascular risk factors (F[2,91] = 1.96, *p* < .01, *partial η*^*2*^ = 0.11). Post-hoc comparisons revealed significant differences in K^trans^ between participants with AD-dementia (K^trans^ = 0.55 × 10^− 3^ min^-1^ ± 0.74 × 10^− 3^ min^-1^), compared to the NC group (K^trans^ = 0.19 × 10^− 3^ min^-1^ ± 0.37 × 10^− 3^ min^-1^; *p* < .01) and the MCI group (K^trans^ = 0.17 × 10^− 3^ min^-1^ ± 0.22 × 10^− 3^ min^-1^; *p* = .03). The MCI group showed no significant differences in K^trans^ values compared to the NC group (*p* = .77; Fig. 3a).

Regarding sPDGFRβ, analysis of variance with and without covariates revealed no significant differences between the cognitive groups (Fig. 3b; *p* = .20). sPDGFRβ was also not significantly different if the cognitive groups were identified solely by neuropsychological testing (*p* = .47).

### AD-pathology and sPDGFRβ/DCE-MRI

**Fig. 4:**
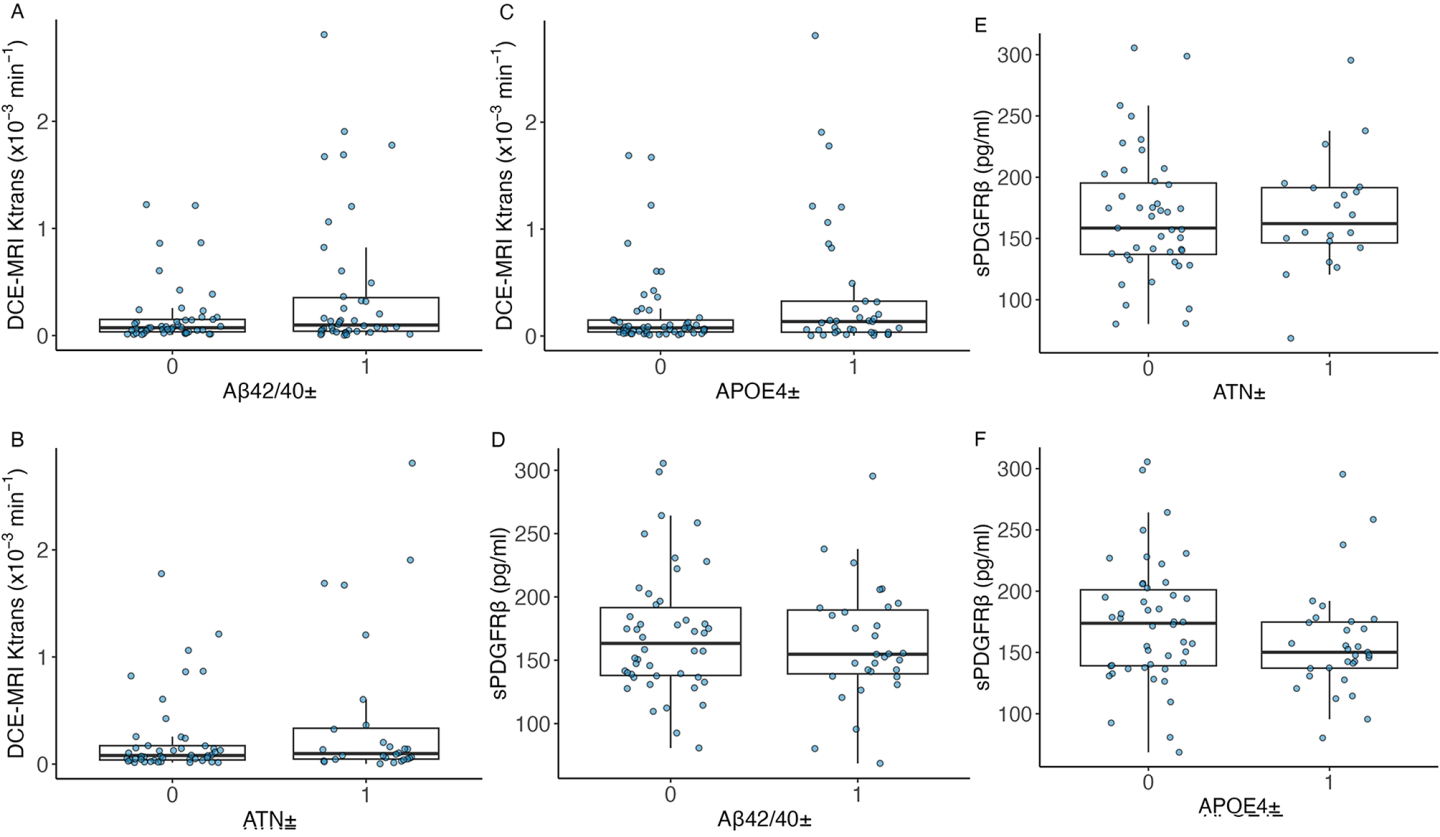
Boxplots depicting no significant differences in Ktrans or sPDGFRβ depending on Aβ42/40-, ATN- or APOE4-status. Significance testing using t-tests. Positivity of standard AD biomarkers was based on local accepted cut-off values. **A-F** failed to reach statistical significance Abbrevations: sPDGFRβ = soluble platelet-derived growth factor receptor β; Aβ = amyloid-beta; APOE = apolipoprotein E gene; ATN: amyloid-beta positivity based on CSF-Aβ42/40, tau positivity based on CSF-p(181)tau, positivity for neurodegeneration based on CSF total tau

**Fig. 5:**
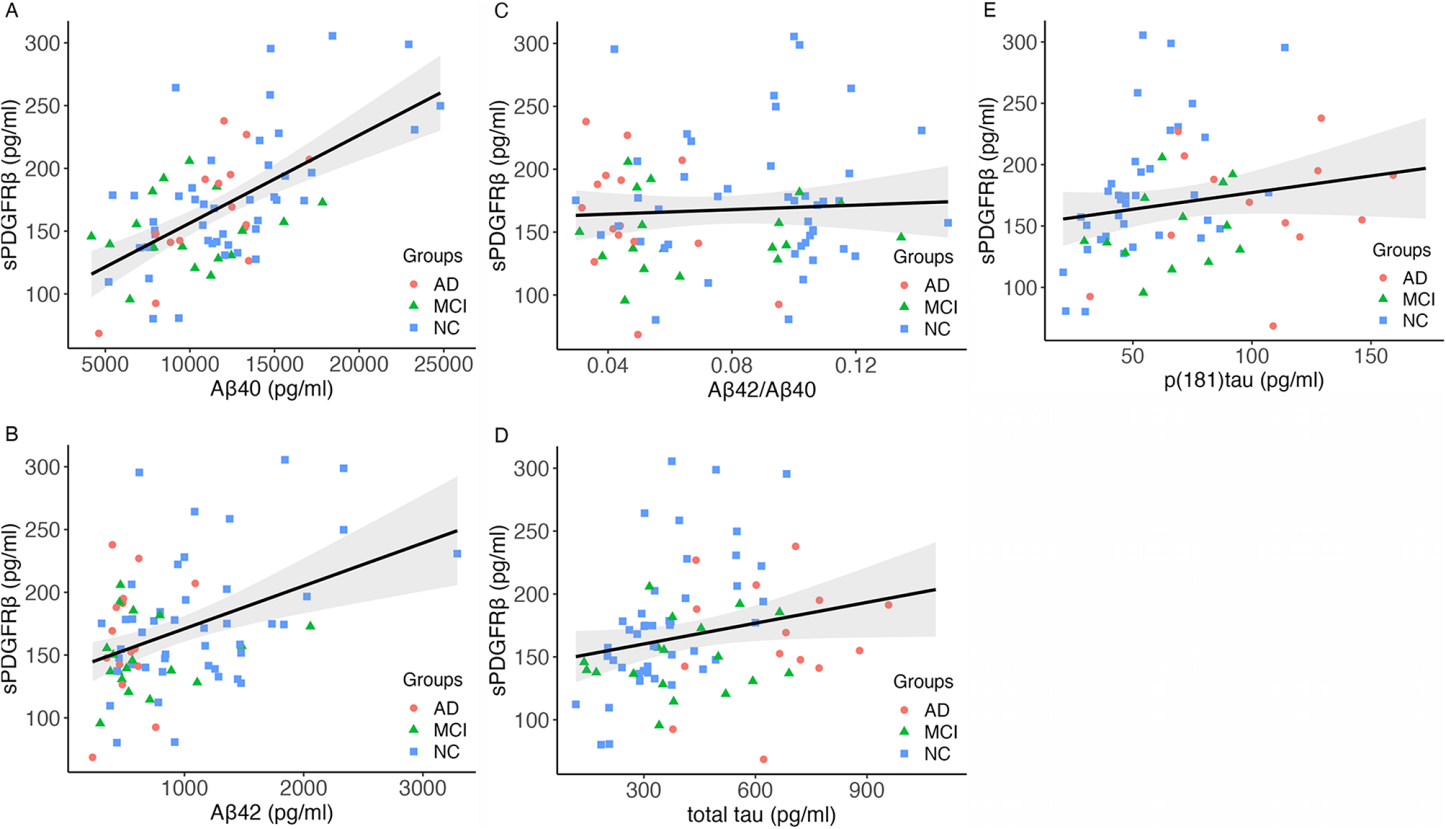
Correlation analyses between sPDGFRβ and ATN biomarkers. A Scatterplots depicting the correlations between sPDGFRβ and with **A** Aβ40 (*r* = 0.57, *p* < .01), **B** Aβ42 (*r* = 0.39, *p* < .01), **C** Aβ42/Aβ40 (*r* = 0.06, *p* = .62), **D** total tau (*r* = 0.30, *p* < .01), **E** p(181)tau (*r* = 0.30, *p* = .02) Abbreviations: Aβ = amyloid-beta; sPDGFRβ = soluble platelet-derived growth factor receptor-β

APOE4-carriers (heterozygote and homozygote combined) did not differ significantly in sPDGFRβ (*p* = .22) or K^trans^ (*p* = .28). sPDGFRβ and K^trans^ was not associated with amyloid-status defined by Aβ40/42 cut-off values (sPDGFRβ: *p* = .43; DCE-MRI: *p* = .19) or overall AD-biomarker positivity (sPDGFRβ: *p* = .82; DCE-MRI: *p* = .44; Fig. 4). We did find significant positive linear associations between sPDGFRβ and Aβ40 (*r =* .57, *p* < .001) and between sPDGFRβ and Aβ42 (*r* = .39, *p* < .001). Furthermore, significant associations between sPDGFRβ and total tau (*r* = .30, *p* < .01) and p(181)tau (*r* = .30, *p* = .018) were observed (Fig. 5).

### Neuroinflammation

**Fig. 6:**
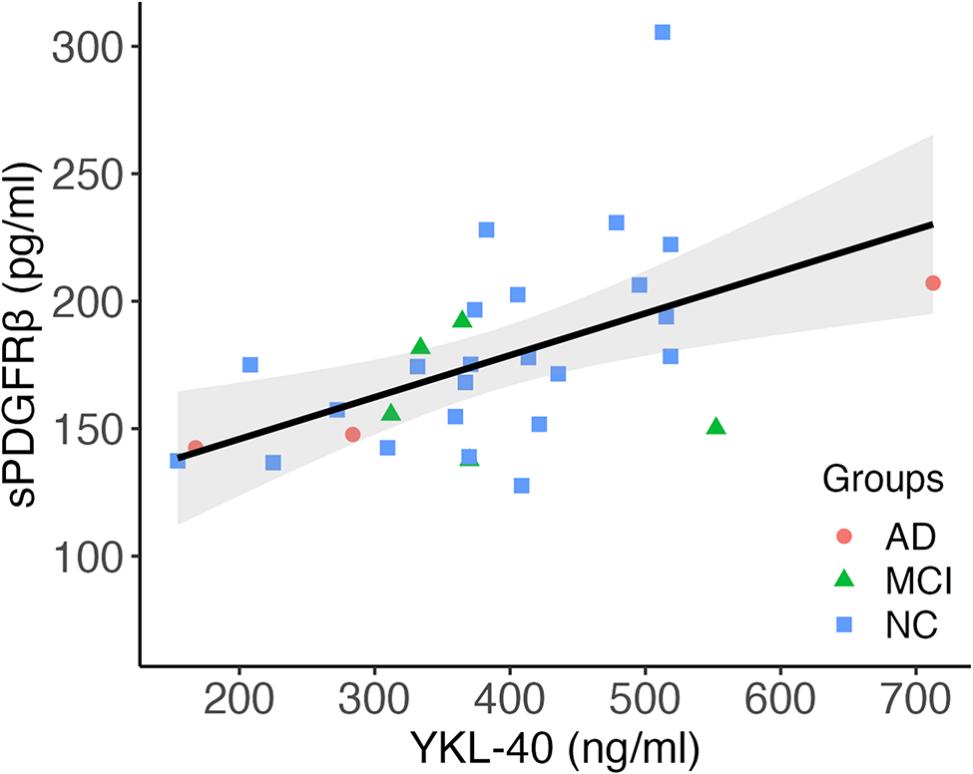
Scatterplot depicting a significant correlation between sPDGFRβ and YKL-40 (*r* = 0.53, *p* < .01) Abbreviations: sPDGFRβ = soluble platelet-derived growth factor receptor β; YKL-40 = chitinase 3-like protein 1

A correlation matrix (Supplementary materials Figure S1) between sPDGFRβ and the panel of neuroinflammatory markers revealed one highly significant correlation between sPDGFRβ and YKL-40 (*r* = .54, *p* < .001; Fig. 6), which remained significant after Bonferroni-correction for multiple testing and remained significant at the standard significance-threshold in a hierarchical linear regression model controlling for age, sex, and APOE4-status (*b* = 0.801, β = 0.461, *p* = .020). K^trans^ did not significantly correlate with any of the neuroinflammatory biomarkers.

## Discussion

Our results revealed BBB disruption in the hippocampus in patients with AD dementia, but not MCI. sPDGFRβ was not associated with cognitive impairment. Instead, sPDGFRβ increased with age independent of vascular risk factors and was linked to increases in YKL-40, a glycoprotein associated with activated astrocytes and neuroinflammation [42]. sPDGFRβ or K^trans^ values were not associated with AD-biomarker-status or APOE4 but sPDGFRβ exhibited a positive correlation with soluble AD biomarkers, particularly demonstrating a robust association with Aβ40 but not markers of amyloid-plaque burden (Aβ42/40).

Whereas several studies have found BBB disruption in the form of hippocampal BBB disruption [25], increases in sPDGFRβ [18] or QAlb [14, 15] to be associated with cognitive impairment, we could only identify BBB disruption using DCE-MRI in the hippocampus of patients with AD-dementia. Hippocampal BBB function was not significantly different between patients with MCI compared to normal cognition. Furthermore, hippocampal BBB dysfunction was not associated with aging or white matter hyperintensities. Hippocampal BBB dysfunction also did not significantly correlate with QAlb thereby possibly indicating that focal BBB changes in the hippocampus of patients with AD may not be significantly associated with overall BBB integrity. sPDGFRβ, which was significantly associated with QAlb also did not correlate significantly with DCE-MRI. Elevations in sPDGFRβ were not discernible in either the MCI group or the AD dementia group, despite the latter exhibiting indications of localized BBB breakdown in the hippocampus.

In line with Cicognola and colleagues [28] we identified age-dependent increases in sPDGFRβ. While aging induces a multitude of changes, like DNA damage [43] and mitochondrial dysfunction [44], Cicognola and colleagues hypothesize that changes in BBB function may be associated with age-dependent increases in neuroinflammation along pericyte degeneration. Due to the small sample size, and the subgroup consisting mainly out of participants in the NC-group, we did not perform elaborate statistical models within this subgroup. The results of our exploratory analysis confirm the reported association between YKL-40 and sPDGFRβ highlighting the intricate involvement of pericytes in neuroinflammatory processes, however. YKL-40 is thought to be an indicator of activated astrocytes [42, 45] and has been observed in neurodegenerative diseases that are linked to neuroinflammation such as Creutzfeldt-Jakob disease [46] but also AD [47]. In previous studies we observed increases in YKL-40 to be associated with elevations in tau protein even in preclinical AD [30]. The role of pericytes in neuroinflammation is complex. Pericytes detect inflammatory states and respond dynamically to it taking on a neuroprotective or a proinflammatory phenotype [48]. They can structurally change in response to inflammation [49] and induce and maintain proinflammatory states through secretion of various cytokines [50]. On the other hand, pericytes can also respond to inflammatory states with detachment of the basal membrane [48] and apoptosis [51]. Pericyte degeneration and subsequent BBB breakdown may then further promote inflammation via the invasion of blood-borne, pro-inflammatory substances such as fibrinogen [8, 9, 52] resulting in a vicious cycle. Our findings notably revealed significant correlations exclusively between sPDGFRβ and YKL-40. This may underscore the potential significance of pericyte-astrocyte interactions within the neurovascular unit, distinct from other inflammatory mediators like IL-6 or CRP.

In our study, soluble Aβ, especially Aβ40 was highly correlated with sPDGFRβ, as well as with YKL-40. Aβ has been linked to induce vasoactive effects in pericytes [53] and is associated with cerebrovascular dysfunction [54] which becomes most evident in cerebral amyloid angiopathy (CAA). In CAA, Aβ40, less prone to aggregation into parenchymal plaques, deposits in cerebral vessels undermining vascular integrity co-leading to microbleeds, lacunes, white matter hyperintensities and resulting in increased perivascular spaces [55]. Aβ has also been linked to detrimental effects directly affecting pericytes. For instance, studies have shown that Aβ may lead to constriction of pericytes [56] and increases sPDGFRβ-shedding significantly [17]. Furthermore, immunohistochemical studies have revealed increased astrocytosis, as measured by increased YKL-40 expression, adjacent to amyloid-plaques and cerebral vessels loaded with Aβ pathology [46]. Lastly, Aβ can also induce neuroinflammation by activating perivascular macrophages [57, 58]. It seems plausible therefore that increases of soluble Aβ may damage pericytes via direct mechanisms but may also underscore the relevance of Aβ for pericyte-dependent inflammatory responses.

As initially proposed by the two-hit hypothesis [5], BBB dysfunction may predispose for amyloid-plaque formation due to a reduction in Aβ clearance. Aβ is cleared out of the CSF by pericytes which internalize Aβ via LRP-1 receptors [6]. Tachibana and colleagues [59] were able to show that pericyte implantation in an amyloid AD mouse model results in reduced amyloid burden. Studies have also reported the expression of BACE-1 within pericytes thereby allowing for the subsequent cleavage of Aβ40 into the smaller peptide Aβ34 [20]. Therefore, the association between sPDGFRβ, and increases in soluble Aβ could also partly reflect pericytes’ reduced Aβ-clearance capabilities. Crucially, however, we could not find any association between sPDGFRβ or DCE-MRI and amyloid-plaque burden as measured by Aβ42/40 or overall AD-biomarker status. APOE4-status has also been hypothesized as a risk factor for BBB-breakdown via activation of the Cyp-A-MMP9 pathway [60, 61] and has been associated with increased hippocampal BBB leakiness and higher sPDGFRβ [26]. Also here, we found no evidence for APOE-status to be associated with BBB changes, analog to Cicognola and colleagues [28] and others [62].

### Limitation

This study has several limitations. Due to the study design, we could not analyze sPDGFRβ, QAlb and the biomarkers of neuroinflammation in the full sample resulting in exploratory subgroup analyses and potentially underpowered statistical testing. Overall, the group sizes were uneven with the NC group being the largest. Accordingly, YKL-40 was predominantly assessed in the NC-subgroup. Therefore, we cannot draw conclusions about its differential association to sPDGFRβ in the context of AD. Furthermore, the markers of BBB function and the markers of neuroinflammation were not assessed at the same study visit. Due to overall sample restriction replication samples were not possible. Since this is a cross-sectional study, causality cannot be inferred. To establish causality, particularly regarding the relationship between amyloid-β and sPDGFRβ, future research should focus on analyzing longitudinal data.

## Conclusion

Our study highlights the intricate relationship between BBB dysfunction, neuroinflammation, and AD biomarkers. While hippocampal BBB disruption was evident in AD dementia, sPDGFRβ levels were primarily associated with age rather than cognitive impairment. The correlation between sPDGFRβ and neuroinflammatory marker YKL-40 suggests a link between pericyte dysfunction and neuroinflammation. Additionally, the association between sPDGFRβ and soluble Aβ underscores a potential mechanism linking BBB dysfunction to soluble Aβ levels. Whether increases in soluble Aβ are caused by reduced pericytes’ reduced clearance capabilities or whether soluble Aβ is associated with pericyte dysfunction due to its vasculotoxic properties remains elusive. Crucially, we could not find evidence that pericyte dysfunction is linked to amyloid plaque burden, overall AD biomarker status or APOE4. Further investigation into these complex interactions including longitudinal observations is required for a more thorough understanding of the relevance of the BBB in AD.

### Electronic Supplementary Material

Below is the link to the electronic supplementary material.

Supplementary Material 1

## Data Availability

No datasets were generated or analysed during the current study.

## Abbreviations

Aβ: Amyloid-β
AD: Alzheimer’s disease
ANOVA: Analysis of variance
APOE: Apolipoprotein E
ARWMC: Age-related white matter change score
AXL: AXL Receptor Tyrosine Kinase
BBB: Blood-brain barrier
C1q, C3, C3b, C4: Complement factors
CDR: Clinical Dementia Rating
CRP: C-reactive protein
CSF: Cerebrospinal fluid
DCE-MRI: Dynamic contrast-enhanced magnetic resonance imaging
GDS: Geriatric Depression Scale
IL: Interleukin
IP-10: Interferon-gamma induced protein 10
MCI: Mild cognitive impairment
MCP-1: Monocyte Chemoattractant Protein-1
MIF: Macrophage Migration Inhibitory Factor
MMSE: Mini-Mental State Examination
NC: Normal cognition
QAlb: Ratio of albumin in cerebrospinal fluid and blood
SNP: Single nucleotide polymorphism
sPDGFRβ: Soluble Platelet-Derived Growth Factor Receptorβ
TREM2: Triggering Receptor Expressed on Myeloid Cells 2
Tyro3: TYRO3 Protein Tyrosine Kinase
YKL-40: Chitinase 3-like protein 1
