# Supplementary material for: Assessing blood-brain barrier dysfunction and its association with Alzheimer’s pathology, cognitive impairment and neuroinflammation
Source: Alzheimers Res Ther. 2024 Jul 31;16:172. doi: 10.1186/s13195-024-01529-1 (PMC11290219; doi:10.1186/s13195-024-01529-1)
Supplement: Supplementary file 1 — Supplementary Material 1 [file 13195_2024_1529_MOESM1_ESM.docx]

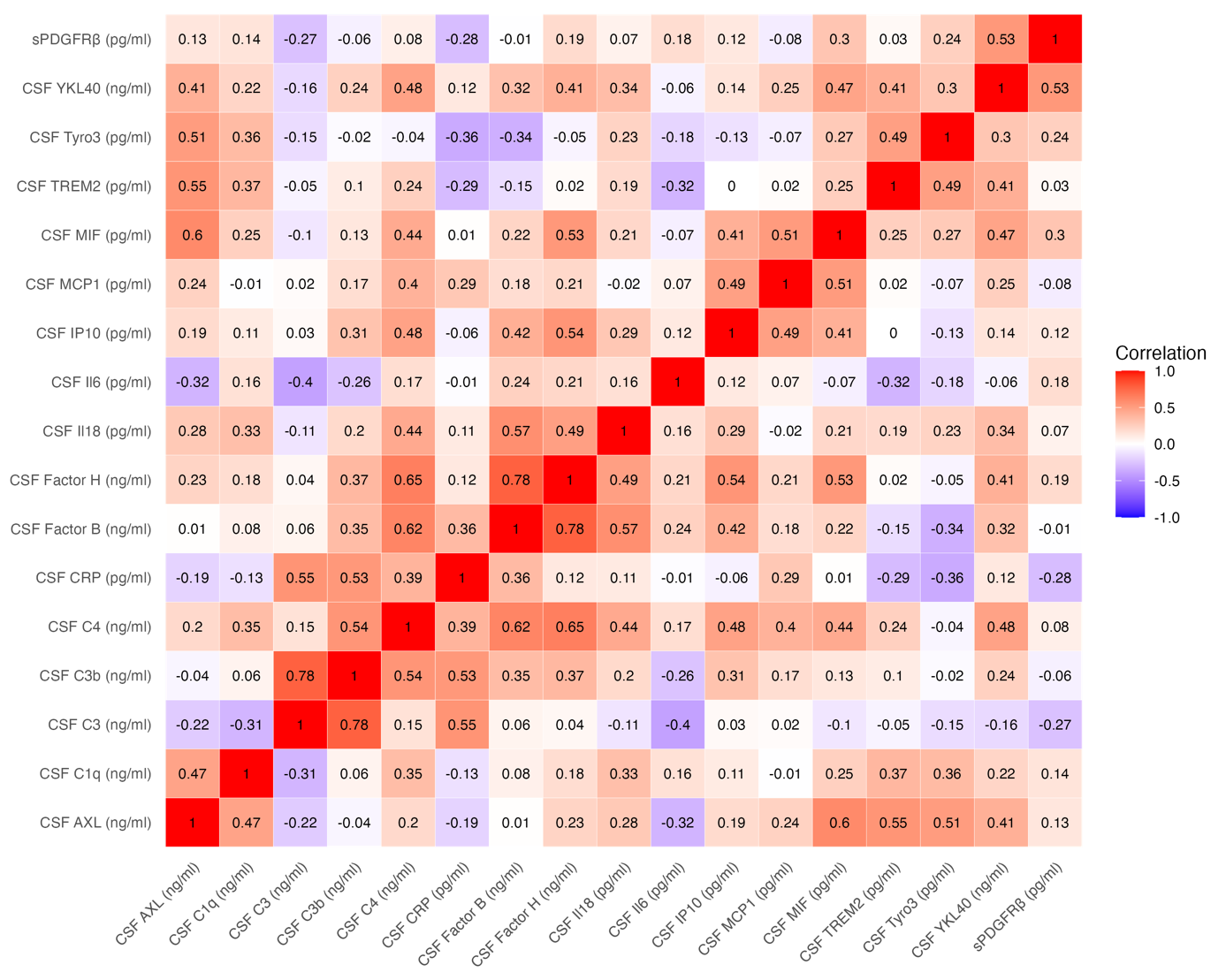


**Figure S1. Correlation matrix depicting the correlations between sPDGFRβ and the panel of neuroinflammatory biomarkers.**

Abbreviations: AXL = AXL Receptor Tyrosine Kinase; C1q = Complement factor C1q; C3 = Complement factor C3; C3b = Complement factor C3b; C4 = Complement factor C4; CRP = C-reactive protein; IL = Interleukin; IP-10 = Interferon-gamma induced protein 10; MCP-1 = Monocyte Chemoattractant Protein-1; MIF = Macrophage Migration Inhibitory Factor; sPDGFRβ = Soluble Platelet-Derived Growth Factor Receptor β; TREM2 = Triggering Receptor Expressed on Myeloid Cells 2; Tyro3 = TYRO3 Protein Tyrosine Kinase; YKL-40 = Chitinase 3-like protein 1.

**
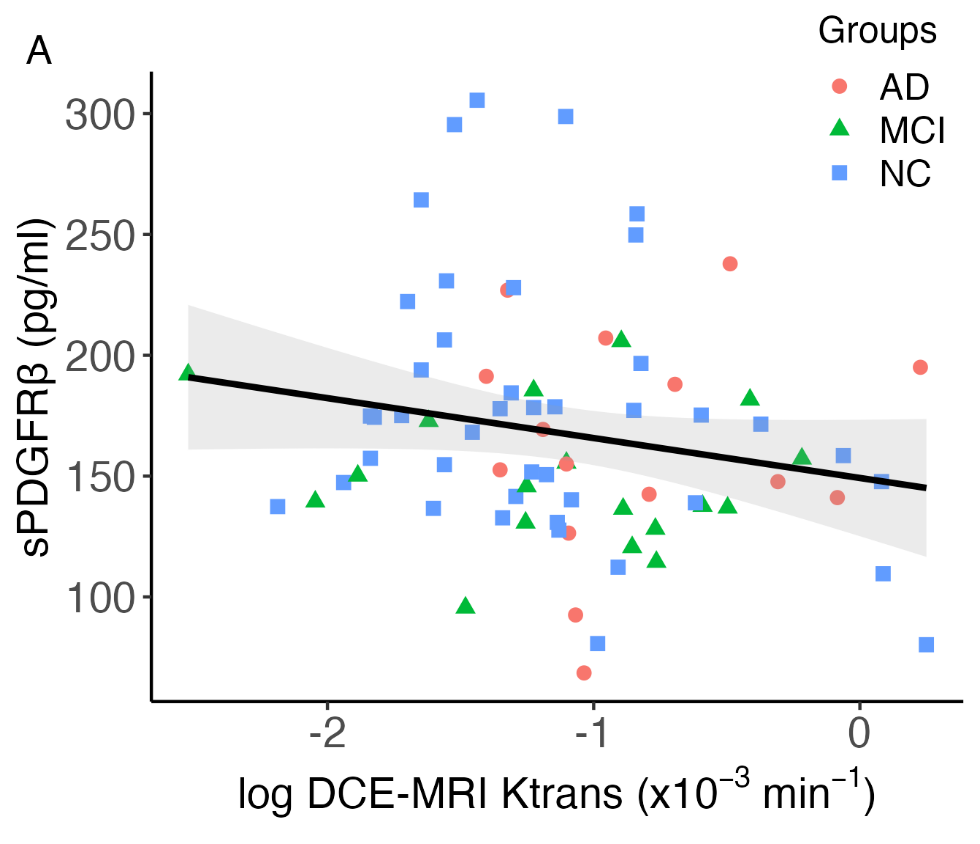
**

**
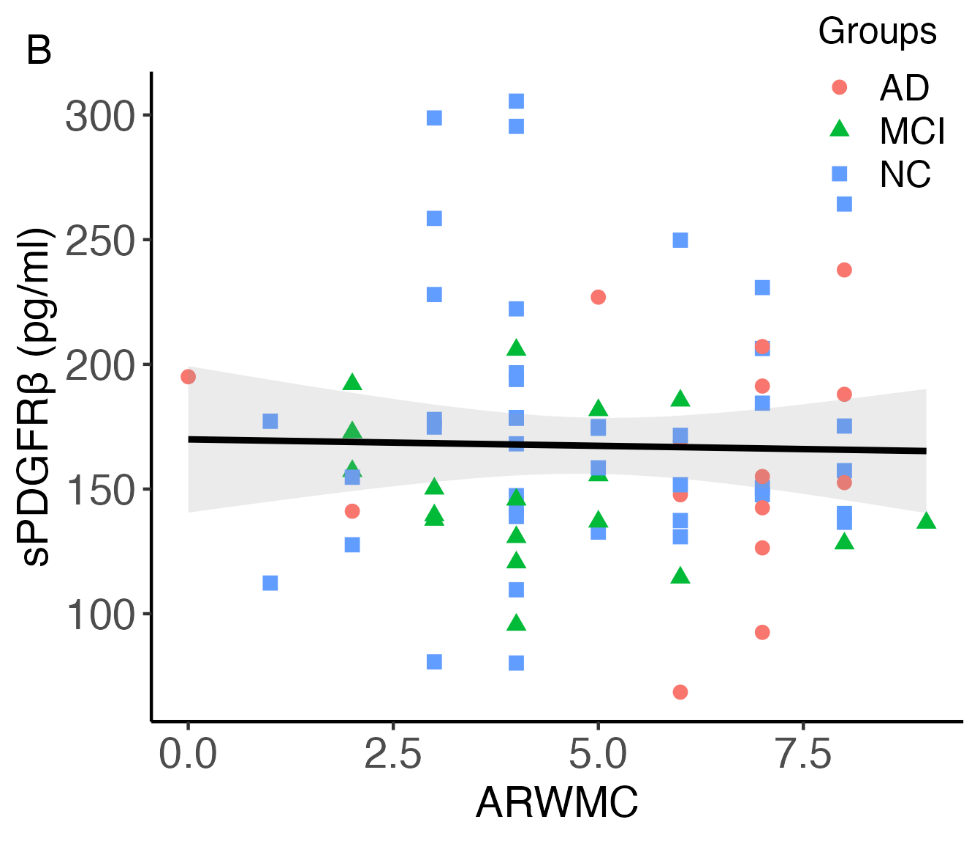
**

**
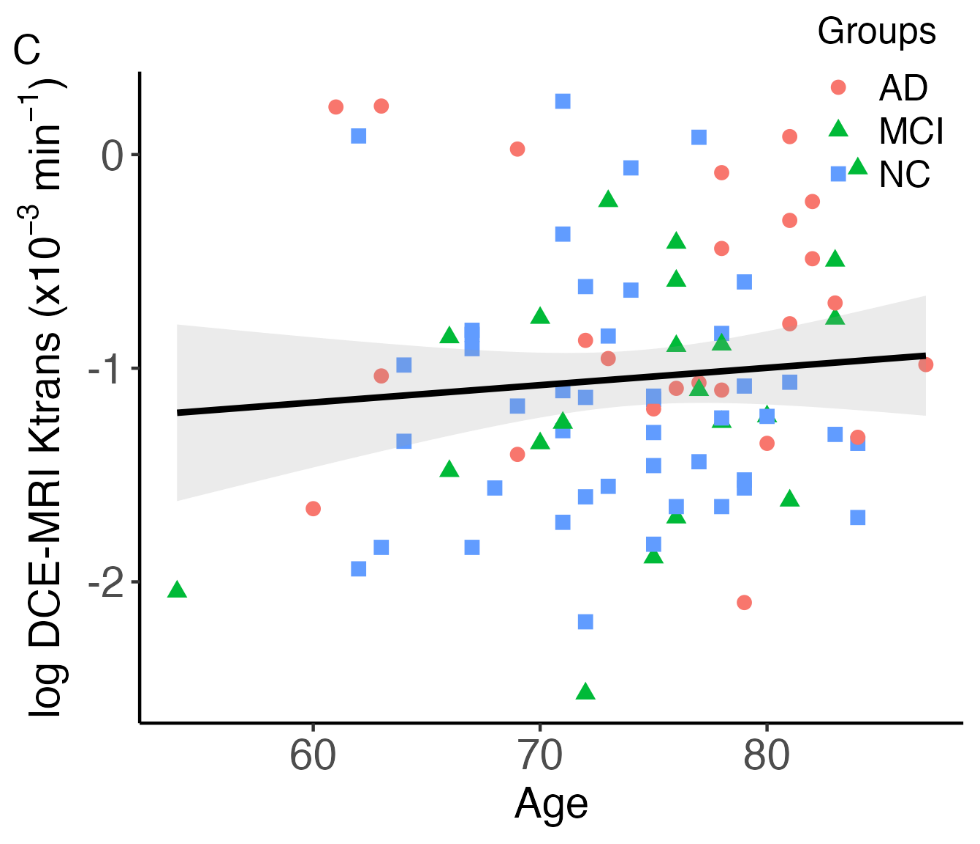

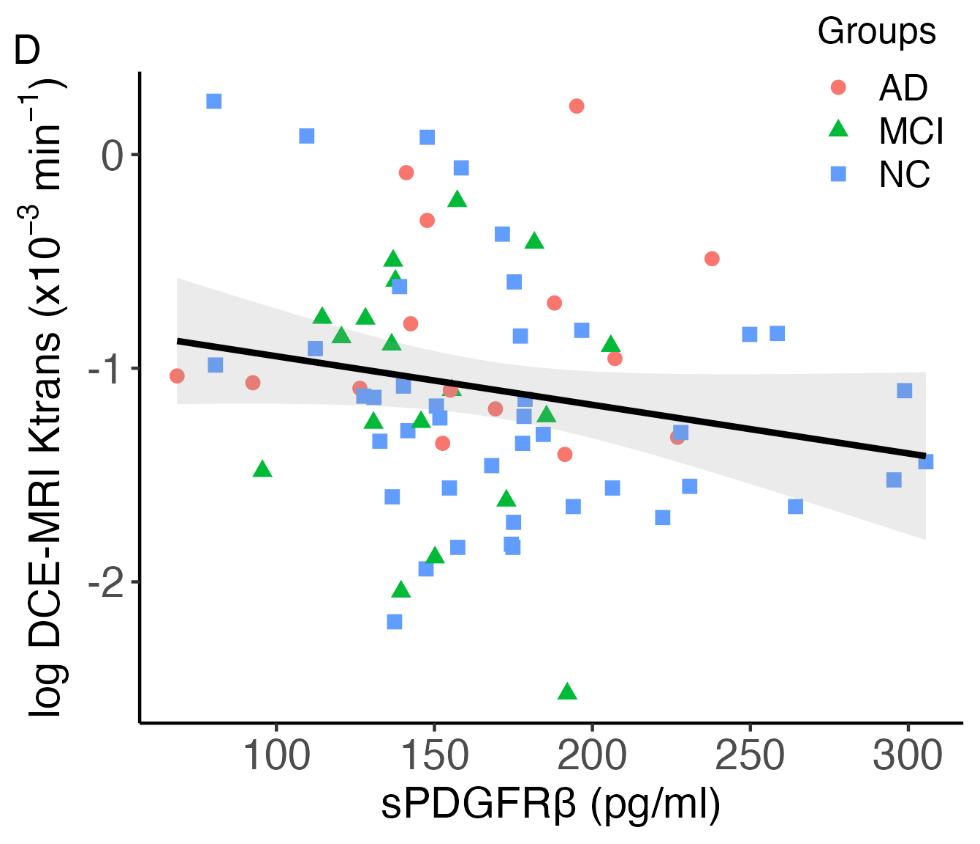
**

**Figure S2. Additional correlation analyses,**  **scatterplots depicting the correlation between** **A** DCE-MRI K^trans^ and sPDGFRβ (*r*=-0.21, *p*=.07), **B** sPDGFRβ and ARWMC (*r*=0.001, *p*=.99), **C** DCE-MRI K^trans^ and age (*r*=0.004, *p*=.97), **D** DCE-MRI k^trans^ and ARWMC (*r*=-0.7*, p*=.49).

Abbreviations: ARWMC = age-related white matter changes; DCE-MRI = dynamic contrast-enhanced MRI; sPDGFRβ = soluble platelet-derived growth factor receptor-β.
